# Complete Genome Sequences *Microbacterium paraoxydans* phages BarnCat, Pochacco, Smelly and SpiderBri and *Microbacterium foliorum* phage Crisis

**DOI:** 10.17912/micropub.biology.001840

**Published:** 2025-12-22

**Authors:** Ana Acosta, Micah Baga, Kallysta Ballweber, Karenna Brand, Yehee Cho, Tanner Cox, Anheli Franco, Felipe Guevara Lopez, Felicity Hernandez, Lia Hutchins, Adam Johnson, Hyeri Kang, John Kang, Hojeong Kim, Mason Kim, Estrella Klar, Yongmin Kwon, Alexis Lee, Noah McMillan, Lauren Michel, Ruth Montiel, Shalom Mugenzi, Layla Murillo, Ethan Nguyen-Khoa, Traysen Nhem, Necker Peralta, Julienne Role, Natallie Ruiz, Annika Samayoa, Bridgette Sanchez, Ahmi Shin, Janelle Terriquez, Mateo Toledo, Zadie Tsao, Andrew Velasquez, Natasha Dean, Arturo Diaz

**Affiliations:** 1 Biology, La Sierra University, Riverside, California, United States

## Abstract

As part of the Science Education Alliance-Phage Hunters Advancing Genomics and Evolutionary Sciences program, we report the discovery and genome sequence of microbacteriophages Crisis, BarnCat, Pochacco, Smelly, and SpiderBri. Phages were isolated from soil samples in Riverside, CA and Viroqua, WI using the hosts *Microbacterium foliorum* and *Microbacterium paraoxydans.*

**Table 1. Phage Genome Information:** Genome characteristics of phages isolated in this study, including host, lysate titers, GC content, genome length, character of genome ends, number of predicted open reading frames (ORFs), number of predicted tRNAs, and phage particle characteristics. Phages were assigned to clusters based on gene content similarity of at least 35% to phages in the Actinobacteriophage database. Sequencing information for each phage as well as sample collection location and the phage isolation method are also listed.

**Table:** 

Phage	Crisis	BarnCat	Pochacco	Smelly	SpiderBri
Isolation host	Microbacterium foliorum NRRL B-24224	Microbacterium paraoxydans NRRL B-14843	Microbacterium paraoxydans NRRL B-24275	Microbacterium paraoxydans NRRL B-24275	Microbacterium paraoxydans NRRL B-24275
Sample location (city, state)	Riverside, CA	Viroqua, WI	Riverside, CA	Riverside, CA	Riverside, CA
GPS coordinates	33.9116 N, 117.50198 W	43.494722 N, 90.863889 W	33.90076 N, 117.49401 W	33.90738 N, 117.50807 W	33.8946 N, 117.51979 W
Isolation method	Enriched	Enriched	Direct	Enriched	Enriched
Lysate Titer (pfu/ml)	4.9 x 10^10	5 x 10^9	5.4 x 10^10	1.66 x 10^10	2.5 x 10^10
Approximate read coverage (fold, x)	90	638	10,921	10,907	4,143
# of 150 base single-end reads	31,873	268,38			
# of 100 base single-end reads			1,408,121	2,000,240	743,422
Genome length (bp)	52,987	61,511	17,880	17,859	17,868
GC content %	68.90%	58.10%	52.70%	52.90%	52.30%
Character of genome ends	Circularly permuted	Direct Terminal Repeat	Covalent Terminal Protein	Covalent Terminal Protein	Covalent Terminal Protein
# of ORFs (# with predicted function)	90 (29)	125 (32)	25 (12)	24 (12)	25 (12)
# of tRNAs	0	1	0	0	0
Capsid size range	71 nm (n=1)	65 nm (n=1)	32-37 nm (n=8)	50-53 nm (n=12)	37-38 nm (n=8)
Tail length range	135 nm (n=1)	174 nm (n=1)	9-10 nm (n=6)	15-17 nm (n=12)	10-11 nm (n=2)
Plaque size range	1-1.6 mm (n=10)	1-3 mm (n=50)	1-1.8 mm (n=10)	0.5-1.2 mm (n=10)	1.4-2.1 mm (n=10)
Cluster Assignment	EC	GB	GK	GK	GK
GenBank accession #	PP750965	PP978804	PV915814	PV15830	PV915808
SRA accession #	SRX30257450	SRX30257449	SRX30257453	SRX30257454	SRX30257455

## Description

Comparative analysis of the genomes of phages has provided important insights into bacteriophage evolution and diversity. To expand the catalog of bacteriophages capable of infecting *Microbacterium,* five phages were isolated from soil samples collected in Southern California and Wisconsin using *Microbacterium foliorum* NRRL B-24224, and two *Microbacterium paraoxydans* species, NRRL B-14843 and NRRL B-24275, as hosts (Table 1) (Jacobs-Sera et al., 2020). Members of the genus *Microbacterium* are Gram-positive and commonly found in diverse environments, including soil, aquatic habitats, dairy products, and plant surfaces (Russell et al. 2019). Certain species, such as *M. foliorum* and *M. paraoxydans* , have also been isolated from clinical sources, including blood specimens and wound swabs (Laffineur et al. 2003; Gneiding et al. 2008).

Soil samples were initially suspended in peptone-yeast extract-calcium (PYCa) liquid medium and incubated at 30° C with agitation for 90 minutes. Following incubation, the mixtures were centrifuged, and the supernatants were filtered using a 0.22 μm membrane (Zorawik et al., 2024). For phages isolated via the "direct" method, 500 μl of each filtrate was mixed with 250 μl of *M. paraoxydans* in PYCa soft agar and incubated at 30° C for up to 48 hours to allow plaque development. In the "enrichment" method, filtrates were inoculated with either *M. paraoxydans* or *M. foliorum* and incubated with shaking at 30° C for 72 hours. These cultures, potentially enriched for microbacteriophages, were subsequently refiltered and plated in soft agar containing the corresponding bacterial host. Clear plaques were observed in all cases, and phages were purified by performing three rounds of plaque picking and plating. For each round of purification, plaques were picked that were at least 1 cm from the nearest plaque neighbor. High-titer lysates were prepared and used for negative-staining transmission electron microscopy (TEM) using uranyl acetate (Zorawik et al., 2024). Based on TEM imaging, phages Crisis and BarnCat exhibited siphoviral morphology, whereas Pochacco, Smelly, and SpiderBri displayed podoviral morphology. Phage lysate titers, capsid width, tail length, and plaque size ranges are listed in Table 1.

The phenol-chloroform DNA extraction protocol was optimized for high-titer lysate samples. Lysates were treated with RNase/DNase and Proteinase K, followed by incubation and phase separation using phenol:chloroform:isoamyl alcohol. DNA was precipitated with sodium chloride and ethanol, washed with 70% ethanol, air-dried, and resuspended in water. DNA quality and concentration were assessed using a NanoDrop spectrophotometer. Library preparation was performed using the NEB Ultra II FS Kit. For phages Crisis and BarnCat, sequencing was conducted on an Illumina MiSeq 1000 (v3 reagents), generating 150 bp single-end reads. For phages Pochacco, Smelly, and SpiderBri, sequencing was conducted on an Illumina NexSeq 1000 (XLEAP-P1 kit), generating 100 base reads. MiSeq raw reads were assembled without trimming whereas the NextSeq raw reads were trimmed with cutadapt 4.7 (using the option: –nextseq-trim 30) and filtered with skewer 0.2.2 (using the options: -q 20 -Q 30 -n -l 50) prior to assembly (Jiang et al., 2014; Martin, 2011). Assembly of raw reads was carried out using Newbler v2.9 with default settings (Russell 2018), and completeness of the assembled genomes was manually assessed using Consed v29 (Gordon and Green 2013). Read counts and estimated genome coverage are detailed in Table 1. The resulting genome lengths ranged from 17,859 to 61,511 bp, with GC contents between 52.3% and 68.9% (Table 1).

Genome annotation was performed using the Phage Evidence Collection and Annotation Network (PECAAN) platform (Rinehart et al., 2016). Gene start and stop sites were predicted using a combination of Glimmer v3.02 (Delcher et al *.* 2007), Genemark v2.5 (Besemer and Borodovsky 2005), and Starterator v.546f (Pope & Jacobs-Sera, 2018). Functional predictions of encoded proteins were determined using a combination of HHpred (PDB_mmCIF70, UniProt, Pfam-A v.36, and NCBI Conserved Domain databases) (Söding et al *.* 2005), BLASTp v.2.16.0 (Altschul et al. 1997) alignments against the Actinobacteriophage protein (https://phagesdb.org/) (Russell & Hatfull, 2017) and NCBI non-redundant protein sequences databases (https://blast.ncbi.nlm.nih.gov), and Phamerator (Cresawn et al *.* 2011). Transmembrane helices were identified using DeepTMHMM v1.0.44 (Hallgren et al *.* 2022), TOPCONS v2.0 (Tsirigos et al. 2015), and SOSUI v1.11 (Hirokawa et al. 1998). tRNAs were detected using ARAGON v1.2.41 and tRNAscan-SE v2.0.12 (Laslett and Canback 2004; Lowe and Chan 2016). All software were used with default parameters.

No integrase or repressor functions could be identified across any of the genomes of these five phages, suggesting that these phages do not undergo lysogeny and are thus predicted to be lytic phages. Phages were assigned to clusters using the phagesDB Gene Content Similarity (GCS) tool, based on a minimum of 35% shared gene content with existing entries in the Actinobacteriophage database (Russell & Hatfull, 2017). Pochacco, Smelly, and SpiderBri are the inaugural members of cluster GK, and the genomes are characterized by the presence of a gene encoding a predicted terminal protein, which functions by covalently attaching to the phage genome termini where it can prime the initiation of DNA replication. Another notable feature shared by all three phages is that the predicted terminase gene is in the middle of the lysis cassette, downstream of two genes encoding lysin–associated domains, specifically an endolysin with a protease M15 domain followed by an endolysin with a protease M23 domain, and upstream of two transmembrane proteins. Additionally, all three have a relatively low GC content compared to most *Microbacterium* species (~52% vs ~67%) (Jacobs-Sera et al., 2020). Crisis was assigned to cluster EC and the genome is circularly permuted. As is the case for all cluster EC phages, the putative open reading frames in Crisis's genome are transcribed unidirectionally, and contain an asymmetric 14-bp sequence (CTATAGGTGTAAGC) upstream of transcriptional start sites (Barruga et al., 2024; Jacobs-Sera et al., 2020; Kim et al., 2022; Lin et al., 2020; Miller et al., 2019). BarnCat was assigned to cluster GB and the end of the genome has direct terminal repeats. BarnCat encodes two putative endonuclease VII proteins, and four HNH endonucleases scattered throughout the genome. In comparison, the cluster GB phages Lifes and WaterT are not predicted to encode any HNH endonucleases, whereas Cassita and LeeroyJenkins encode one and two, respectively. Among the cluster GB phages, LeeroyJenkins is the only other phage predicted to encode a putative endonuclease VII.

**Data Availability**

GenBank and Sequence Read Archive (SRA) accession numbers are provided in Table 1.
